# Exploring data management content in doctoral nursing handbooks

**DOI:** 10.5195/jmla.2021.1115

**Published:** 2021-04-01

**Authors:** Rebecca Raszewski, Abigail H. Goben, Martha Dewey Bergren, Krista Jones, Catherine Ryan, Alana Steffen, Susan C. Vonderheid

**Affiliations:** 1 raszewr1@uic.edu, Associate Professor & Information Services & Liaison Librarian, Library of the Health Sciences Chicago, University of Illinois at Chicago, Chicago IL; 2 agoben@uic.edu, Associate Professor & Data Management Coordinator & Liaison Librarian, Library of the Health Sciences Chicago, University of Illinois at Chicago, Chicago, IL; 3 bergren@uic.edu, Clinical Professor, Clinical Professor, Associate Department Head, Health Systems Science, Director, Advanced Population Health Nursing, Health Systems Leadership & Informatics, University of Illinois—Chicago College of Nursing, Chicago, IL; 4 kjones29@illinois.edu, Director, Urbana Regional Campus, Clinical Associate Professor Department of Health Systems Sciences, University of Illinois—Chicago College of Nursing, Champaign, IL; 5 cryan4@uic.edu, Clinical Associate Professor, Department of Biobehavioral Health Science, University of Illinois—Chicago College of Nursing, Chicago, IL; 6 steffan@uic.edu, Senior Biostatistician, Research Assistant Professor, Department of Health Systems Sciences, University of Illinois—Chicago College of Nursing, Chicago, IL; 7 vonde@uic.edu, Clinical Assistant Professor, Department of Women, Children, and Family Health Science, University of Illinois—Chicago College of Nursing, Director of Nursing Research, University of Illinois Hospital & Health Sciences System, Chicago, IL

**Keywords:** data life cycle, data management, nursing doctoral education, student handbooks

## Abstract

**Objective:**

While data management (DM) is an increasing responsibility of doctorally prepared nurses, little is understood about how DM education and expectations are reflected within student handbooks. The purpose of this study was to assess the inclusion of DM content within doctoral nursing student handbooks.

**Methods:**

A list of 346 doctoral programs was obtained from the American Association of Colleges of Nursing (AACN). Program websites were searched to locate program handbooks, which were downloaded for analysis. A textual review of 261 handbooks from 215 institutions was conducted to determine whether DM was mentioned and, if so, where the DM content was located. Statistical analysis was performed to compare the presence of DM guidance by type of institution, Carnegie Classification, and the type of doctoral program handbook.

**Results:**

A total of 1,382 codes were identified across data life cycle stages, most commonly in the handbooks’ project requirements section. The most frequent mention of DM was in relation to collecting and analyzing data; the least frequent related to publishing and sharing data and preservation. Significant differences in the frequency and location of codes were identified by program type and Carnegie Classification.

**Conclusions:**

Nursing doctoral program handbooks primarily address collecting and analyzing data during student projects. Findings suggest limited education about, and inclusion of, DM life cycle content, especially within DNP programs. Collaboration between nursing faculty and librarians and nursing and library professional organizations is needed to advance the adoption of DM best practices for preparing students in their future roles as clinicians and scholars.

## INTRODUCTION

Better understanding of data and facility in its use by doctorally prepared nurses has become critical over the last decade. As described by Westra et al., the push for data science and big data research across the field of nursing has become prevalent in nursing research and specifically in the established field of nursing informatics [1]. The latest American Association of Colleges of Nursing (AACN) Essentials explicitly recognizes the importance of data as part of current nursing education and practice [2], emphasizing the need to be prepared to use all sorts and sizes of data to make decisions for operations and patient care. Federal agencies have increased the expectations for those receiving funding for doctoral studies. The Agency for Healthcare Research and Quality recently announced the first National Institutes of Health agency requirement for data management plans (DMPs) for grant applicants [3]. With this increased focus on gathering, analyzing, and applying data, it is critical that data management (DM) become a foundational component of nursing education for doctoral nursing programs. Data management is defined as “the process of validating, organizing, securing, maintaining, and processing scientific data, and of determining which scientific data to preserve” [4].

Although DM has been explored in the health sciences library literature, it has focused primarily on asking faculty across health sciences disciplines about their DM needs [5] or preparing health sciences librarians to provide DM services within their institutions [6]. Librarians recognize the increased importance of data as scholarly output [7], and their expertise in information management has the potential to meet emerging DM needs [8]. The literature on these early stages of offering DM services, however, suggests that few health sciences librarians have considered how DM is currently being addressed in nursing curricula. One exception is McGowan et al., who described a case study introducing DM as a component of evidence-based practices for undergraduate nursing [9]. Building on a rich history of librarian and nursing faculty collaborations, such as those related to evidence-based practice within hospital settings [10–19], nursing education [20–29], and public health nursing outreach [30], DM is a new opportunity for partnership.

A further limitation is that DM education has received little attention in nursing literature regarding doctoral programs. Articles on DM are mostly case studies describing course development related to clinical DM [31–33] or using data mining to determine patterns in DNP patient-student encounters and end-of-program competencies [34]. Our survey of DM education in nursing doctoral programs explored how nursing curricula are educating students to work with their final projects or dissertation data through all stages of the data life cycle. However, that study focused on methods of instruction and current practices and did not comprehensively address recommended DM practices or resources provided by the nursing college/department or institutions for their students [35]. Academic student handbooks are a standard resource that provide information on policies and program requirements for students. Although research has been conducted using student handbooks, the focus has been almost entirely on undergraduate nursing instruction, with limited attention paid to DM [36–39]. In both nursing and health sciences literature, little research has analyzed the content of graduate handbooks to determine the policies related to DM in doctoral programs.

The purpose of this study, therefore, was to examine the inclusion of the DM life cycle stages within nursing doctoral program handbooks. If programs are including DM in their handbooks, we sought to determine whether the handbooks are addressing topics across the data life cycle and to identify the section in the handbooks where DM is described, such as in nursing college/department policies, courses, or dissertation or project requirements for graduation. Additionally, we sought to describe any variance between Doctor of Nursing Practice (DNP) and Doctor of Philosophy in Nursing (PhD) program handbooks.

## METHODS

### Identification of doctoral nursing program handbooks

A list of DNP and PhD programs from a 2016 AACN list of institutions conferring nursing doctoral degrees was compiled into a spreadsheet [40]. The PhD programs included the Doctor of Nursing Science and Doctorate in Nursing Education. Some institutions from this list were members of a consortium that collaborated in offering a doctoral program. We decided to list each institution from a consortium separately, in case each institution had different student handbooks for the doctoral program. Each institution was assigned a numerical identifier and was searched on the Carnegie Classification website [41] to record its Basic classification category and whether it was a public, private, or for-profit institution. The Carnegie Classification categorizes academic institutions in the United States based on factors such as research expenditures, public versus private, degrees granted, number of full-time students, etc., allowing for institutional comparison.

The list of programs originally included 346 institutions. Eight institutions were eliminated because they were not currently offering a nursing doctoral program or were not accredited by the Commission on Collegiate Nursing Education, the accrediting body for DNP programs. The websites of the remaining 338 nursing colleges and departments were searched to locate the most recently published edition of student handbooks available between April 2017 and February 2018. Nursing handbooks were located by browsing the student-focused sections of the nursing program websites or by searching for nursing handbooks on the entire website. Supplemental handbooks or materials were also downloaded if they contained information on DNP or PhD student requirements. A total of 277 handbooks and supplemental documents (81.9%) of the targeted colleges/department were located. The handbooks were coded using Dedoose [42], a qualitative and mixed-methods data analysis tool.

### DM extract coding in doctoral nursing handbooks

Codes for the handbooks were developed based on the UK Data Service Research Data Lifecycle [43]. The following codes were used: Analyzing Data; Collecting Data; Planning for DM; Preserving Data; Processing Data; Publishing and Resharing Data; and Reusing Data. The Analyzing and Processing stages were split into two separate codes because we wanted to distinguish between the actual processing of data versus data analysis.

Handbooks were also coded based on the context or location of where DM fell within the handbooks. The following codes were used: College Policy; Competencies; Compliance; Course Name or Description; Project Requirements; and Other. A “Great Examples” code was created to highlight unique or exemplary instances of DM within the handbooks. A complete list of codes and their definitions is provided in Appendix 1. After the codes and institutional descriptors were uploaded into Dedoose, the handbooks were uploaded and affiliated.

To locate DM content within the handbooks, the word *data* was used to search the handbooks. As most of the handbooks (which were in PDF format) could not be searched within Dedoose, they were searched separately using Adobe Acrobat. If a mention of *data* was relevant to DM, that particular section of the handbook was coded in Dedoose with the applicable data life cycle code and the location of the DM. Multiple DM codes could be applied to a relevant reference to DM within a handbook. For example, a course description that mentioned collecting and analyzing data would be coded with both corresponding codes. However, only one location code was used for each section.

Inter-rater reliability was used to assess the consistency of coding. Twenty-three handbooks (8%) were selected at random [44] by independent coders, and it was determined the coding was consistent, with 100% agreement.

The following mentions of data in handbooks were excluded: patient care situations; student data capture for American Databank; library subscription databases; administrative data collection related to clinical hours; or DM unrelated to the doctoral program. Handbooks without relevant mentions were coded as data not mentioned, DM not included, or DM not relevant.

### Statistical analysis

The 277 handbooks and supplemental documents were reduced to 261 unique handbooks with codes summed across multiple documents where appropriate. Summary codes were aggregated across location and lifecycle codes and used for comparisons by Carnegie Classification, public versus private, and DNP, PhD, and combined DNP/PhD handbooks. We used descriptive statistics, independent samples t-tests, chi-squared statistics, and analysis of variance with Tukey's Honestly Significant Difference post-hoc tests to evaluate differences.

## RESULTS

### General characteristics of nursing doctoral student handbooks

Two hundred sixty-one handbooks were analyzed: 28 from institutions that combined handbooks for both DNP and PhD programs, 176 from DNP programs, and 57 from PhD programs. Thirteen programs had handbooks with multiple documents, which were concatenated for our analysis. There were 215 institutions represented in our dataset (46 had two handbooks). One hundred sixty-six institutions were public, 90 were private not-for-profit, and five were not in the Carnegie Classification or were for-profit. One hundred forty-six institutions were classified as doctoral with moderate, high, or very high research activities, while 115 did not have research classifications as determined from their Carnegie Classification Ranking [41].

### Data management in nursing doctoral student handbooks

DM-coded extracts were present in 179 (69%) of doctoral handbooks; 82 handbooks did not mention DM across any aspect of the research data lifecycle. Of the handbooks that did not mention DM, 66 were DNP handbooks, 8 were PhD handbooks, and 8 were combined DNP/PhD handbooks.

**Figure 1:**
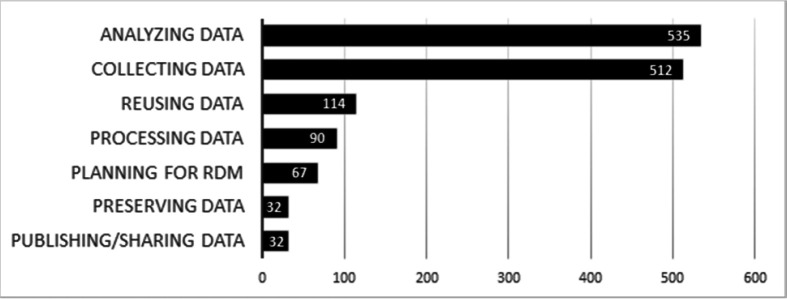
Data management life cycle codes

A total of 1,382 DM-coded extracts based on the UK Data Service Research Data Lifecycle stages were identified across the evaluated handbooks. The average number of extracts in a handbook was 5.17 (SD = 6.37), with a range of 0 to 37. When arranged by life cycle stage, Analyzing Data (535 extracts) and Collecting Data (512 extracts) were each five times more frequent than the next most common code, Reusing Data (114). Other life cycles stages were found in fewer than 100 extracts each (Processing Data: 90; Planning for DM: 67; Preserving Data: 32; and Publishing and Sharing Data: 32). Figure 1 illustrates the total number of codes used for the data life cycle stages mentioned in the doctoral handbooks.

**Figure 2:**
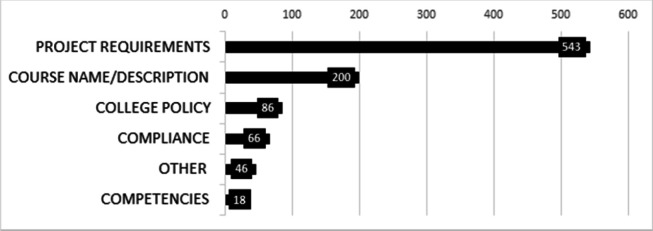
Location of data management within handbooks

The location of the data within the handbooks was analyzed. A total of 959 DM location-coded extracts were identified across the evaluated handbooks. Project Requirements had the highest number of codes (543), followed by Course Names and Descriptions (200). Other codes were found in fewer than 100 extracts each (College Policy: 86; Compliance: 66; Other: 46; and Competencies: 18). Figure 2 illustrates the total number of codes used to indicate where DM was located within the handbooks.

### DM handbook representation by institution classification and type

When analyzing the handbooks by their Carnegie Classification and type of institution, the five handbooks that were not in the Carnegie Classification or were for-profit were combined with those of private, non-profit institutions. Handbooks were sorted into two broad categories by Carnegie Classification: doctoral institutions included Carnegie doctoral-granting institutions with moderate, higher, or highest research activity, while non-doctoral institutions included Carnegie master's colleges, special focus four-year, and baccalaureate colleges. Handbooks retrieved from institutions that had a Carnegie Classification of doctoral-granting with moderate or higher ranking had a significantly higher count of DM-coded extracts in their handbooks than non-doctoral institutions (non-doctoral institutions’ mean = 3.8; SD = 5.5; doctoral-granting institutions’ mean = 6.2; SD = 6.8; *t*(259) = −3.2, *p* = .0016). Close to half (42%) of the non-doctoral-granting institutions had no mention of DM. Just under a quarter (23%) of doctoral institution handbooks had no DM extracts (χ^2^(1) = 10.16, *p* = .0014). Handbooks from institutions with a doctoral-granting Carnegie Classification had a significantly greater number of locations where DM-coded extracts were mentioned throughout the handbook (non-doctoral institutions’ mean = 2.8; SD = 3.9; doctoral granting institutions’ mean = 4.4; SD = 4.5, *t*(259) = −3.07, *p* = .0023). There was not a significant difference between private non-profit institutions and public institutions in the number of codes across the data life cycle (*t*(254) = −0.07, *p* = .9469) or the number of locations in the handbook (*t*(254) = −0.60, *p* = .5498).

An ANOVA test was performed and showed mean differences in the representation of DM activities depending on the type of doctoral handbook (DNP, PhD, combined DNP/PhD; F(2, 258)=16.85, *p* < .0001). Post-hoc tests revealed significant differences at the .05 level, showing that the mean for DNP handbooks (3.7; SD = 5.0) was lower than the means for PhD handbooks (8.7; SD = 7.1) and combined DNP/PhD handbooks (7.3; SD = 8.8). This demonstrates that there was a significant difference in how frequently programmatic handbooks address DM throughout the data life cycle. Further statistical analysis was performed to assess whether there was a difference between handbook type in relation to where DM-coded extracts were found within the handbooks. The means and standard deviations for the number of locations within handbooks are as follows: DNP handbook (2.7; SD = 3.7), combined DNP/PhD handbooks (4.3; SD = 4.2), and the PhD handbooks (6.2; SD = 5.1). A statistically significant difference was found for DM life cycle information locations in the three types of handbooks (F(2, 258)=16.35, *p* ≤ .0001), and Tukey post-hoc tests showed a higher mean number of locations in PhD handbooks compared to DNP handbooks (*p* < .05).

### DM handbook representation by doctoral program type

#### Great examples

The full list of handbooks that were coded with “Great Examples” are listed in Table 1 along with the corresponding DM life cycle stage(s). Most of the great examples from nursing PhD programs focused on preserving or reusing data. Several PhD programs provided recommendations, resources, or policies for preserving student data. Michigan State University had its own Data Archive Rules and Regulations policy. The Michigan State College of Nursing set up a data warehouse that contains all primary research data and datasets used for secondary data analysis. Loma Linda University advised that “Students must adhere to the University Policy on collection, storage, and use of research data,” even including links to university data storage policies [45]. This handbook also included information on how long data collected by their school of nursing personnel or students should be stored. The University of Oklahoma included safe data practices in collecting and storing student research data files. The University of Arkansas for Medical Sciences’ handbook was one of the few to list guidelines regarding ownership of data and authorship of published manuscripts. For reusing data, Boston College provided recommendations for students using existing data or specimens toward their dissertations, while Emory University included a data use agreement example within their handbook. The University of Utah may have been the only handbook that included a section on the use of patents and restricted data for dissertations.

**Table 1:** Great examples in programs and handbooks

Program Name (Type)	Handbook Website	DM Life Cycle Stage
Boston College (PhD)	https://www.bc.edu/bc-web/schools/cson/sites/students.html#phd	Reusing Data
Emory University (PhD)	http://www.nursing.emory.edu/audience-guides/students-audience-guide.html	Reusing Data
Fairleigh Dickinson University (DNP)	http://view2.fdu.edu/academics/university-college/school-of-nursing-and-allied-health/academic-programs/doctor-of-nursing-practice-d-n-p/	Analyzing Data Collecting Data Processing Data
Florida International University (PhD)	https://cnhs.fiu.edu/resources/students/handbooks-manuals/index.html	Collecting Data
Georgia State University (DNP)	https://lewis.gsu.edu/files/2020/01/DNP-Handbook-2019-2020-FINAL.pdf	Publishing and Resharing Data Reusing Data
Johns Hopkins University (DNP/PhD)	https://nursing.jhu.edu/academics/resources/catalogue.html	Analyzing Data Collecting Data Planning for DM Preserving Data Processing Data
King University (DNP)	https://www.king.edu/programs/nursing/dnp/	Collecting Data Preserving Data Processing Data Publishing and Resharing Data
Loma Lima University (PhD)	https://nursing.llu.edu/sites/nursing.llu.edu/files/docs/nursing-PhDHandbook1416.pdf	Preserving Data
Medical University of South Carolina (DNP/PhD)	https://nursing.musc.edu/academics	Preserving Data Processing Data
Michigan State University (PhD)	https://nursing.msu.edu/student-resources/handbooks	Preserving Data Processing Data Reusing Data
The Ohio State University (DNP)	https://nursing.osu.edu/students/student-resources/student-handbooks	Analyzing Data Collecting Data Reusing Data
Saint Mary's College (DNP)	https://grad.saintmarys.edu/sites/default/files/DNPStudentHandbook.pdf	Collecting Data
Southern Illinois University (DNP)	https://www.siue.edu/nursing/academic-programs/graduate/pdf/STUDENT-HANDBOOK-FINAL-2017-2018.pdf	Collecting Data Processing Data Reusing Data
University of Arkansas for Medical Sciences (PhD)	https://nursing.uams.edu/	Analyzing Data Collecting Data Planning for DM Preserving Data Publishing and Resharing Data Reusing Data
University of Kentucky (DNP/PhD)	https://www.uky.edu/nursing/academic-programs-ce/academic-resources/student-handbooks	Analyzing Data Collecting Data Planning for DM
University of Louisville (DNP)	https://louisville.edu/nursing/academics/dnp/dnp-resources	Processing Data Preserving Data
University of Massachusetts Lowell (PhD)	https://www.uml.edu/Health-Sciences/Nursing/Programs/Doctoral/phd/default.aspx	Analyzing Data
University of New Mexico (PhD)	https://hsc.unm.edu/college-of-nursing/education/student-affairs/student-handbooks.html	Analyzing Data Reusing Data
University of Oklahoma (PhD)	https://nursing.ouhsc.edu/Current-Students/Student-Handbooks	Collecting Data Preserving Data
University of Utah (PhD)	https://nursing.utah.edu/programs/graduate/phd/	Reusing Data
Wichita State University (DNP)	http://nursing.utah.edu/programs/pdfs-handbooks/phd_policy_progression.pdf	Preserving Data

For the great examples that applied to DNP programs, a broader range of DM life cycle codes were used compared to the PhD handbooks. Johns Hopkins University was the only university that had courses coded as great examples. This handbook listed a series of courses on clinical DM that were essential for the evaluation of any “Evidence-Based Practice/Performance Improvement project” [46]. The courses integrate DM skills such as analyzing, collecting, and cleaning data. The University of Kentucky's mention of a DM contact for students may have been the only such instance in any of the handbooks. Wichita State University was one of the few DNP programs that said students should include their DNP projects’ data file when submitting their final project. There were also examples from other DNP handbooks that touched on reusing data. Georgia State University recommended that students ask if the primary investigator has a data-use agreement for the student to sign for reusing data. King University, the Medical University of South Carolina, and Southern Illinois University provided questions to consider or recommendations on deidentifying data. Ohio State University's handbook contained a process that their students must follow for accessing, collecting, and/or using existing data at their medical center.

## DISCUSSION

This research project examined the presence of DM content in doctoral nursing program handbooks. This handbook analysis revealed significant variation in how DM is addressed in these standard documents and suggested opportunities for improvement.

### Outcomes of handbook analysis

Across the handbooks reviewed, data collection and analysis were the most common codes applied to extracts. This was not surprising, as these parts of the data life cycle are the activities expected of all doctoral nursing students [47]. What it suggests, however, is that many nursing programs or faculty may not consider data collection and analysis in the context of a larger data life cycle and are not yet providing guidance for the other aspects of the DM life cycle and giving them appropriate weight.

Our research found more mentions of DM across the data life cycle and across the entire handbook for PhD handbooks compared to combined PhD-DNP or DNP handbooks. This is likely correlative with the expectation that PhD students are more likely to generate novel data. However, it potentially ignores the fact that DNP students will be working not only with data they capture for their capstone projects but also with data in electronic health records and other sources they use to monitor quality and safety in the clinical setting. The statistically greater frequency and variation of extracts in PhD handbooks could be related to the programs’ longer duration or the training of the faculty teaching within the programs. The DNP program is relatively new, yet it has increased exponentially from 20 programs in 2006 to 348 programs in 2018 [48–49]. Improved guidance from professional DNP educational organizations is needed to accelerate the inclusion of DM requirements in the DNP handbooks.

While no significant difference was found between public and private institutions, there was a difference between handbooks from doctoral programs at R1 and R2 research institutions compared to those from doctoral programs at institutions that did not have a Carnegie Classification. This suggests that institutions that have a research focus and emphasis consider DM more comprehensively, including for education and expectations of doctoral nursing programs. It is possible that this also correlates with the already existing resources available to assist and support doctoral nursing students in successfully meeting DM requirements.

### Drawing upon the great examples codes

The rarity of the exemplar codes, a total of 35 marked across 21 handbooks, was a surprise to us and speaks to the inconsistency with which DM is addressed in doctoral nursing student handbooks. Where great examples existed, they did not cover all stages of the data life cycle. The examples that existed were mostly unique to each program identified, suggesting there is not yet an emergent trend of considering DM broadly or targeting DM beyond passing mentions of collecting and analyzing data. Further, the examples showed a lack of guidance or policies for students in preserving or reusing data. There was a lack of examples or forms for students to refer to in situations such as developing a DMP, best practices for storing data properly, or what should be in place to preserve their data before they graduate. Where examples could be identified, most came from handbooks of programs at larger research-focused institutions where DM support and knowledge of obligations may be more pervasive. The examples highlighted here do not comprehensively reflect the entire DM life cycle but are examples that faculty and librarians can refer to when reviewing their college/department handbooks for recommendations or potential areas of collaboration.

### Application for librarians

When developing an understanding of programs with which they are liaising or planning instruction, librarians draw on handbooks for a wealth of information about program expectations, courses, student obligations, and priorities. The handbooks are also regularly updated, so they serve as a place where librarians can recommend DM best practices to be integrated and to introduce resources. This can be done by the library's liaison to the nursing program, either individually or in coordination with a DM librarian or specialist, as is relevant to the institution.

Librarians should recommend, and assist nursing doctoral programs in adding recommendations about, the entire DM life cycle in graduate handbooks, moving beyond data collection and analysis to comprehensively prepare DNP and PhD students for their future research and DM responsibilities. These recommendations could include providing suggested content, such as definitions of the data life cycle stages and activities and resource contacts within the nursing college/department as well as the library or the institution more broadly, as appropriate.

Depending on the DM activities provided by the library and the staffing available, specific required guidance to meet with a librarian to review a DMP or pursue data sharing options could be an additional recommendation. Librarians and nursing faculty could collaborate to develop a list of questions to guide students in collecting and managing data for their projects or research. They may also work on developing recommendations for formatting student project data and especially for storing data before and after students graduate. Overall, the DMP should be mandated for all DNP projects and PhD dissertations because it is an example of a best practice and because students will be expected to be proficient in DM in their future roles as expert clinicians and scholars.

## LIMITATIONS

Although this project includes handbooks from most doctoral nursing programs, there are limitations. Discovery of handbooks was hampered by website design and the practice of some institutions of placing handbooks behind institutional logins. Further, while handbooks are a nearly universal document, they are not expected to be exhaustive. There is also no standard format or required content for student handbooks, and some handbooks are more extensive than others. Additional research is recommended to identify other documents and resources that doctoral nursing programs may use as supplemental guidance to their students. Further, the *AACN Essentials* was just updated and the *Association of College and Research Libraries Information Literacy Competency Standards for Nursing* is currently undergoing revision. These revised documents may include more guidance for DM education, policy, and procedures, but if these standards are presented without mechanisms for implementation, doctoral nursing programs may continue to provide insufficient guidance. It likely will be necessary for national library and nursing educational associations to partner to teach DM best practice and integrate the standards into handbooks and accelerate the adoption of DM policies nationwide.

## CONCLUSION

DM has become a common component of health sciences librarianship and an expectation for all doctorally prepared nurses, whether DNPs or PhDs. However, little research to date has explored whether DM as a competency is expected of doctoral nursing students. Program handbooks serve as a central resource for most doctoral programs; they provide direction to students about the requirements for their courses, research and other types of projects, course materials and expectations, and resources. Due to the critical nature of these handbooks, the presence of DM within a handbook suggests whether emphasis or value is placed on DM at each stage of the data life cycle.

Presently, DM life cycle stages are reflected in doctoral nursing handbooks; however, the emphasis is very narrowly on data collection and analysis. To meet the increasing need for all doctorally prepared nurses to manage data competently, librarians can partner with nursing faculty to increase content about DM in coursework, policy, procedures, and student handbooks. Additionally, librarians and their professional organizations can collaborate with nursing organizations to identify comprehensive best practices and advance adoption across institutions.

## Data Availability

Data associated with this article will be made available through the authors’ institutional repository on the figshare platform: https://doi.org/10.25417/uic.13271489.v1.

## SUPPLEMENTAL FILES

**Appendix 1:** Codes and Definitions
