# Supplementary material for: Exploring data management content in doctoral nursing handbooks
Source: J Med Libr Assoc. 2021 Apr 1;109(2):248–57. doi: 10.5195/jmla.2021.1115 (PMC8270346; doi:10.5195/jmla.2021.1115)
Supplement: Supplementary file 1 — Appendix 1: Codes and Definitions [file jmla-109-2-248-s01.docx]

**Appendix One**

**Codes and Definitions**

**Data Management (DM) Lifecycle Codes**
Based on the stages of the UK Data Service Research Data Lifecycle

- **Planning for Research Data Management**-Developing a data management plan. Includes planning for research and DNP projects.
- **Collecting Data**-Collecting data for research and DNP projects.
- **Analyzing Data**-Using statistical software or other related programs for analyzing data.
- **Processing Data**-Cleaning and documenting data.
- **Preserving Data**-Backing up and storing data within the college, university, by the student, etc.
- **Publishing and Sharing Data**-Where and how data will be shared from a research or DNP project is published, etc.
- **Reusing Data**-Secondary data analysis, reusing data from another source such as government data, faculty research, etc.

**Location of Data Management in Handbook**
The context for where data management falls within the handbook. Where and how is data management being used in relation to the DNP or PhD projects?

- **Competencies**-Data management-related skills are mentioned within what students should be able to do by the time they graduate.
- **Compliance**-IRB/Human Subjects, university, ethics (falsification).
- **College Policy**-A policy related to data management but not related to the students' final projects.
- **Course Name or Description**-The name of a course and/or a description of a course that includes one or more of the data lifecycle stages.
- **Project Requirements**-Data management is mentioned regarding a PhD student research project required for graduation or for a DNP student final project for graduation.
- **Other**-Examples of DM that did not fall under the categories above.

**Handbooks Excluded**Reasons why handbooks are not being included in the analysis

- **Data Not Mentioned in the Handbook**-no mentioning of data at all in handbook.
- **Data Management Not Included**-The word data may appear, but it is not related to any aspect of DM. Examples include "Library databases"; "Student data for learning analytics"; "program data”, etc.
- **Data Management Not Relevant**-Data management would appear in the handbook but it was not relevant to the DNP or PhD program.

**Other Codes**

- **Great Examples**-unique examples of DM that were mentioned in handbooks.
